# Synchronous primary cancers of the thyroid and breast: A case report and review of the literature

**DOI:** 10.3892/ol.2014.2625

**Published:** 2014-10-17

**Authors:** LI LIU, JING SHI, FENGFENG MAO, JINLI WEI, DEYUAN FU, JIAXIN ZHANG

**Affiliations:** Department of Thyroid and Breast Surgery, Clinical Medical College of Yangzhou University, Subei People’s Hospital of Jiangsu Province, Yangzhou, Jiangsu 225001, P.R. China

**Keywords:** synchronous neoplasms, breast cancer, thyroid cancer, mechanism, diagnose

## Abstract

The current report presents the case of a 41-year-old female exhibiting synchronous primary cancers of the thyroid and breast. Pathological examination of a tissue sample following biopsy identified papillary carcinoma of the thyroid and invasive ductal carcinoma of the breast to provide a definitive diagnosis of synchronous primary tumors. The patient underwent a modified radical mastectomy and total thyroidectomy. Following regular adjuvant chemotherapy with cyclophosphamide (800 mg), doxorubicin (100 mg) and paclitaxel (120 mg), once every three weeks for 3.5 months, oral levothyroxine and endocrinotherapy was recommended. Two years after the initial diagnosis, the patient was healthy with no disease recurrence. To the best of our knowledge, no association has been identified between the etiology and diagnoses of the two synchronous primary tumors. Thus, the aim of the current report was to improve the understanding of synchronous primary tumors of the thyroid and breast by presenting a review of the associated literature regarding breast and thyroid cancer. The mechanisms of synchronous neoplasms have only recently been elucidated, however, misdiagnosis is common. Clinicians are, therefore, advised to carefully examine patients with thyroid or breast cancer to avoid an incorrect or misdiagnosis. Furthermore, the present report aims to provide a reference for the cancer database, since the majority of analyses of rare diseases are derived from case reports. To improve the understanding of synchronous primary cancers of the thyroid and breast, an analysis of recent studies regarding the underlying mechanisms of synchronous primary cancers was also undertaken.

## Introduction

Since the association between synchronous neoplasms of breast cancer (BC) and thyroid cancer was elucidated in 1966 (1), clinical surgeons have become more familiar with synchronous primary tumors of the thyroid and breast, however, misdiagnosis is common between these two types of cancer. A total of 8.7% of patients with thyroid cancer were diagnosed with breast cancer between 1973 and 1994 in the USA (1,2). It has been reported that the risk of developing BC in females with a history of thyroid carcinoma is higher than that in females with no history of cancer (1–3). Furthermore, the Surveillance, Epidemiology, and End Results database (http://seer.cancer.gov/) have reported that premenopausal women, aged between 20 and 49 years old, with an history of thyroid carcinoma, exhibit a significantly increased risk of developing subsequent breast carcinoma, when compared with females with no history of thyroid cancer (2). Kim *et al* (3) reported that the breast was the most common site of secondary cancers following a primary thyroid cancer. Previous studies have enabled clinical surgeons to be more familiar with synchronous primary tumors of the thyroid and breast. However, misdiagnosis remains common between these two types of cancers. This may be due to difficulties in combining basic research with clinical practice. The aim of the present study was to investigate the association between synchronous primary cancers, which may lead to a lower rate of misdiagnosis of synchronous primary cancers of the thyroid and breast, clinically. In the present study, the case of synchronous primary cancer of the thyroid and breast were reported and the underlying mechanisms of synchronous primary cancers have been elucidated. Written informed consent was obtained from the patient.

## Case report

In March 2012, a 41-year-old female was admitted to Subei People’s Hospital of Jiangsu Province (Yangzhou, China) following the diagnosis of invasive BC at Yizheng City People’s Hospital (Yizheng, China). The patient had undergone a local biopsy five days prior to admission to Subei People’s Hospital of Jiangsu Province, where further diagnosis and therapy was undertaken. The patient was healthy prior to diagnosis and the family history was non-contributory.

Upon admission, the size of the primary biopsy tumor was 2.0×1.5×1.0 cm and the pathological diagnosis was an invasive carcinoma of the breast (that had been determined at the previous hospital). However, details of the initial surgical procedure were unknown. Gross examination revealed a 4.0-cm incision scar that was 1.5 cm above the right nipple. A tumor measuring 3.0×2.5×2.0 cm was identified in the right side of the thyroid lobe, however, no further abnormalities were identified. Preoperative imaging included an ultrasound of the neck, which revealed a hypoechoic heterogeneity in the right lobe of the thyroid (size, 3.2×1.5 cm) exhibiting punctate calcification. The patient was negative for clinical neck lymph node metastasis. A general physical examination did not indicate any abnormalities. A final preoperative diagnosis of invasive carcinoma of the right breast, as well as thyroid cancer was established.

The patient underwent a modified radical mastectomy and total thyroidectomy following the diagnosis of papillary thyroid cancer (PTC), which was determined via intraoperative frozen section evaluation. Subsequently, postoperative pathological examination of the thyroid sample established a diagnosis of papillary thyroid microcarcinoma (longest diameter, 3 mm). As one of the eleven axilla lymph node samples that was removed during the modified radical mastectomy demonstrated metastases, the patient commenced adjuvant chemotherapy with cyclophosphamide (800 mg), doxorubicin (100 mg) and paclitaxel (120 mg), which was administered every three weeks for 3.5 months. Furthermore, oral levothyroxine was administered to maintain thyroid hormone homeostasis. Due to positive progesterone and estrogen receptor (ER) immunostaining of the tumor tissue sample, endocrinotherapy was proposed for a minimum duration of five years. The patient was healthy at the two-year follow-up subsequent to the initial diagnosis.

## Discussion

Synchronous primary tumors are rare, however, epidemiological studies of multiple primary tumors are currently ongoing (1–5). A population-based retrospective cohort analysis conducted by Chen *et al* (2) identified that females with a history of thyroid carcinoma have an increased risk of developing BC, particularly premenopausal Caucasian females (1,3). Although, the presence of BC did not increase the risk of thyroid cancer (2). Furthermore, a previous case-control study (4) revealed that a history of any thyroid disorder or the resultant treatment was not associated with an increased risk of developing BC, however, parous females with a history of thyroid cancer did exhibit an increased risk of developing BC. Thus, according to the above-mentioned literature, a history of thyroid carcinoma may increase the risk of BC. Furthermore, Murray *et al* (5) reported that BC was the most common type of primary non-thyroidal cancer associated with the subsequent development of PTC and that primary non-thyroidal cancer PTC patients presented at a more advanced stage. A previous study indicated that radiotherapy to treat cancer patients may increase the risk of developing a second malignancy (3). The breast and the thyroid are endocrine organs, which are involved in hormonal responses. Thus, it is proposed that the mechanism of these synchronous primary tumors is associated with an interaction between the breast and thyroid hormonal responses.

A literature search was conducted with PubMed (http://www.ncbi.nlm.nih.gov/pubmed), using the terms thyroid, breast and mechanism and the search was limited to studies published in the past five years. The literature search resulted in 11 studies (6–17) and the data is summarized in Table I. The majority of synchronous primary cancer studies focus on the thyroid hormone and propose mechanisms for the activation of the associated oncogene. The studies predominantly emphasized the importance of hormones associated with the thyroid (6–11). For example, one study demonstrated that thyroid hormone receptor-β mutation promotes the development of mammary hyperplasia via the activation of signal transducer and activator of transcription 5 (8). Furthermore, studies regarding the hormones in close proximity to the thyroid, which may be involved in the sychronous primary tumor mechanism, such as thyroid hormone (9), thyroid peroxidase antibody (10) and thyroid Ras-regulating enzymes (11), have been undertaken. The sodium iodide symporter (NIS) was proposed as a potential co-passageway for thyroid and BC (12,13), indicating a potential therapeutic target during radioiodine therapy. Therefore, the specific signaling pathways regulating NIS activity require investigation and a method to upregulate NIS must be determined. Tyrosine kinase inhibitors that are currently adopted for tumor therapy may enhance NIS activity and facilitate radioiodine therapy (12), therefore, the present study proposes that tyrosine kinase inhibitors may serve as a therapeutic target for BC. However, pituitary tumor-transforming gene-binding factor (PBF) was demonstrated to repress NIS, acting as a proto-oncogene (13). Thus, a balance between PBF downregulation and NIS repression is required to maximise radioiodine uptake and improve the prognosis for patients with cancer.

Furthermore, nuclear protein 1 (14), retinoid-inducible nuclear factor (15) and nuclear receptor coactivator amplified in breast cancer 1 (16) were also identified as oncogenic coactivators and potential biomarkers for thyroid cancer and BC. Rearranged during transfection/papillary thyroid carcinoma (RET/PTC) kinase, an oncogene implicated in the tumorigenesis of thyroid cancer, has been identified as an estrogen-dependent gene whose expression is induced by estrogen in BC cells. Furthermore, RET/PTC kinase is a critical regulator in the proliferation of ER-positive BC cells. In addition to the factors presented in Table I, the risk of developing synchronous primary cancers of the thyroid and breast is associated with Cowden and Cowden-like syndrome (18), as well as other hereditary diseases. In the present case the patient has no family history of such diseases, therefore, details of hereditary-associated mechanisms are not provided.

When determining a diagnosis, the clinical surgeon must avoid an under- or misdiagnosis, as the signs and symptoms of metastatic tumors are similar to those of synchronous primary tumors. An evaluation of the prevalence of a second malignancy in patients with an initial diagnosis of thyroid or BC revealed that female thyroid cancer patients had a 0.67-fold increase in the prevalence of a subsequent BC; whereas males had a 29-fold increase, and female BC patients had a two-fold increase in the prevalence of thyroid cancer, whereas males had a 19-fold increase (19). Thus, following treatment of primary breast or thyroid cancer, follow-up and screening may be appropriate. Surgeons should be aware of the frequency of synchronous PTC and consider evaluation of the neck during the diagnosis of non-thyroidal malignancies, particularly for patients being diagnosed with breast, prostate, melanoma or renal cell carcinoma (5). The present case was promptly diagnosed due to the pronounced mass in the anterior region of the neck. Contrast-enhanced ultrasound and microvessel density were identified as efficacious optical methods for evaluating the microcirculation of thyroid cancer in Chinese females with BC (20) and immunohistochemical staining of tissue sections has improved the differential diagnosis in these types of lesion. A previous study identified that patients initially diagnosed with non-thyroidal cancer present with PTC tumor characteristics that are similar to patients with no history of cancer (5), therefore, treatment should be the same for PTC patients whether they have a history of cancer or not. As the presence of thyroid disease did not appear to affect the prognosis of females with BC (21), the present report proposes modified radical mastectomy.

In conclusion, synchronous primary cancers of the thyroid and breast may be associated by endocrine hormone interactions and congenetic oncogenes, which may serve as novel targets for cancer prevention and therapy. Furthermore, emphasis should be placed on determining the correct diagnosis. Since synchronous primary cancers of the thyroid and breast are relatively rare, various factors are required to differentiate between the types of synchronous primary tumor, including the common risk factors, and diagnostic and treatment strategies.

## Figures and Tables

**Table I:** Underlying mechanisms of breast and thyroid cancers.

First author (ref.)	Model	Primary factor	Mechanism	Significance
Kim WG (6)	Mice	*TRβ*	Reactivation of silenced *TRβ* gene expression delays thyroid tumor progression.	Applied to BC.
Ling Y (7)	Patients	*TRβ*	Hypermethylation of *TRβ* as a gene silencing mechanism is prevalent in BC.	Methylated *TRβ* serves as a tumor suppressor and biomarker in BC.
Guigon CJ (8)	Mice	*TRβ*	*TRβ* mutation promotes the development of mammary hyperplasia via STAT5 activation.	Confers a fertile genetic ground for tumorigenesis.
Sar P (9)	I*n vitro* MCF-7 cells	TH	TH induces apoptosis in MCF-7 cells and is associated with downregulation of *SMP-30* gene expression.	Therapeutic target in BC.
Muller I (10)	Patients	TPOAb	TPOAb-positive females with BC have a better prognosis than TPOAb-negative females with BC.	Protective role in BC and an antigenic link between BC and TC.
Carrera-González MP (11)	Rats	Thyroid Ras-regulating enzymes	In rats with mammary tumors, a change of thyroid Ras-regulating enzymes alone or alterations of other types of regulation, such as the hypothalamus-pituitary axis.	Further carcinogenic process.
Gaertner FC (12)	Mice	NIS	Functional active NIS leads to iodide uptake in different types of cancer, including BC.	Radioiodine treatment target in BC.
Smith VE (13)	Patients, Mice	PBF	PBF is pregulated in multiple endocrine tumors, by estrogen, and mediates cell invasion and represses NIS.	Proto-oncogene, a target for improving radioiodine uptake.
Chowdhury UR (14)	Human	NUPR1	NUPR1 facilitates the establishment of metastases and is key in the progression of BC and TC.	Biomarker of cancer.
Knappskog S (15)	Patients	RINF	High RINF expression is associated with poor overall survival in BC. replacing the TP53 mutation as an oncogene of BC.	Unfavorable prognostic factor in BC.
Liu MY (16)	Patients	AIB1	Upregulation of AIB1 in PTC correlates with lymph node metastasis.	Oncogenic coactivator and biomarker for TPC.
Wang C (17)	BC cell lines	RET/PTC kinase	Estrogen-dependent gene expression, induced by estrogen in BC cells; a critical regulator for the proliferation of ER-positive BC cells.	Prognostic biomarker and therapeutic target for ER-positive BC.

TRβ, thyroid hormone receptor β; BC, breast cancer; STAT5, signal transducer and activator of transcription 5; MCF-7, Michigan Cancer Foundation-7; TH, thyroid hormone; SMP-30, senescence marker protein-30; TPOAb, thyroid peroxidase antibody; TC, thyroid cancer; NIS, sodium/iodide symporter; PBF, pituitary tumor-transforming gene-binding factor; NUPR1, nuclear protein 1; RINF, retinoid-inducible nuclear factor; TP53, tumor protein 53; AIB1, (nuclear receptor coactivator) amplified in breast cancer 1; TPC, thyroid papillary cancer; RET/PTC, rearranged during transfection/papillary thyroid carcinoma; ER, estrogen receptor.
